# The Effect of Depth on Drag During the Streamlined Glide: A Three-Dimensional CFD Analysis

**DOI:** 10.2478/v10078-012-0044-2

**Published:** 2012-07-04

**Authors:** Maria L. Novais, António J. Silva, Vishveshwar R. Mantha, Rui J. Ramos, Abel I. Rouboa, J. Paulo Vilas-Boas, Sérgio R. Luís, Daniel A. Marinho

**Affiliations:** 1 University of Trás-os-Montes and Alto Douro. Department of Sports, Health and Exercise, UTAD, Vila Real. Portugal.; 2 Research Centre in Sports, Health and Human Development, CIDESD, Portugal.; 3 University of Beira Interior. Department of Sport Sciences, UBI, Covilhã. Portugal.; 4 University of Trás-os-Montes and Alto Douro. Department of Mechanical Engineering, UTAD, Vila Real, Portugal.; 5 University of Porto. Faculty of Sport, FADEUP, Porto, Portugal.; 6 Porto Laboratory of Biomechanics, Porto, Portugal.

**Keywords:** biomechanics, swimming, performance, simulations

## Abstract

The aim of this study was to analyze the effects of depth on drag during the streamlined glide in swimming using Computational Fluid Dynamics. The Computation Fluid Dynamic analysis consisted of using a three-dimensional mesh of cells that simulates the flow around the considered domain. We used the K-epsilon turbulent model implemented in the commercial code Fluent^®^ and applied it to the flow around a three-dimensional model of an Olympic swimmer. The swimmer was modeled as if he were gliding underwater in a streamlined prone position, with hands overlapping, head between the extended arms, feet together and plantar flexed. Steady-state computational fluid dynamics analyses were performed using the Fluent^®^ code and the drag coefficient and the drag force was calculated for velocities ranging from 1.5 to 2.5 m/s, in increments of 0.50m/s, which represents the velocity range used by club to elite level swimmers during the push-off and glide following a turn. The swimmer model middle line was placed at different water depths between 0 and 1.0 m underwater, in 0.25m increments. Hydrodynamic drag decreased with depth, although after 0.75m values remained almost constant. Water depth seems to have a positive effect on reducing hydrodynamic drag during the gliding. Although increasing depth position could contribute to decrease hydrodynamic drag, this reduction seems to be lower with depth, especially after 0.75 m depth, thus suggesting that possibly performing the underwater gliding more than 0.75 m depth could not be to the benefit of the swimmer.

## Introduction

Swimming is characterized by the intermittent application of a propulsive force to overcome a velocity-dependent water resistance (i.e., hydrodynamic drag - F_D_) (Marinho et al., 2009a). Hydrodynamic drag is the force that a swimmer has to overcome in order to maintain his movement through water and it is influenced by velocity, shape, size and the frontal surface area (Kjendlie and Stallman, 2008; Morais et al., 2011). If, on one hand, the propulsive force is one of the main swimmer’s skills which gathers technical abilities and physical qualities, on the other hand, minimizing the drag force is, by no means, less important. However, it is seen as less dependent on the technique and more influenced by constitutional factors than the first, thus more stable (Vilas-Boas et al., 2001).

The total swimming time is made up of the starting time, swimming time, turning time and finish time (Guimarães and Hay, 1985; Haljand and Saagpakk, 1994), which makes it hard to quantify to what extent each one of them contributes to the final result (Sanders et al., 2006). However, the underwater phases of swimming after starts and turns are a large and important component of the total event time in modern swimming (Marinho et al., 2009b) and can play an important role in the final performance in swimming races (Lyttle et al., 2000; Marinho et al., 2009c). During this phase, the two determining factors of glide performance are the initial push-off velocity and the hydrodynamic drag which decelerates the swimmer. Minimizing drag could produce better results than merely increasing the effort during wall push-off since it does not increase the metabolic cost (Lyttle et al., 1998).

There are two main types of F_D_: the first, known as passive drag, which is the force needed to move the swimmer in a certain and stable position (Kolmogorov et al., 1997; Vilas-Boas et al., 2001); the second, known as active drag (di Prampero et al., 1974; Hollander et al., 1986; Kemper et al., 1976; Kolmogorov and Duplisheva, 1992; Ungerechts, 1994), which aims to assess the F_D_ intensity the swimmer undergoes when swimming freely (Vilas-Boas et al., 2001), i.e., when the amount of drag force is associated to the arm and leg movements performed by the swimmer (Kolmogorov et al., 1997; Lyttle et al., 2000).

When gliding, the swimmer notices the passive drag (D_p_), which is caused mainly by the shape and size of the body and the velocity and depth of the glide.

One method applied to measure swimmer resistance in water is to tow subjects at various velocities (Karpovich, 1933; di Prampero et al., 1974; Jiskoot and Clarys, 1975). However, not all these studies analyzed the drag experienced underwater. Jiskoot and Clarys’s (1975) study was the first that analyzed the drag experienced by swimmer underwater. However, the results reported by them are not in agreement with previous fluid dynamics studies of streamlined objects. These results showed drag was greater immediately under the water surface than at a depth equivalent to a depth-to-length ratio of 0.2 to 0.4 (Hertel, 1966; Larsen et al., 1981) whereas Jiskoot and Clarys (1975) showed that the passive drag experienced by swimmers at 0.6m underwater averaged 20% higher than that recorded at the surface.

Though glide has been the subject of several experimental studies (Lyttle et al., 1999; 2000; Ugolkova, 1999; Goya, 2003), difficulties in quantifying the flow characteristics around the human body render it difficult to estimate depth effects from the hydrodynamic theory (Lyttle et al., 1998). Computational fluid dynamics (CFD) solves and analyzes flow problems through numerical simulations, being an alternative and a complement to experimental procedures, which are sometimes difficult to apply as the analysis of underwater passive gliding.

Thus, the purpose of this study was to determine the effect of depth in total drag during hydrodynamic glide, using CFD. It was hypothesized that hydrodynamic drag decreases with depth during gliding.

## Material & Methods

### Three-dimensional model

In order to create a three-dimensional digital model computer tomography scans of a human body of an Olympic swimmer were applied. With these data we converted the values into a format that could be read in Gambit, Fluent^®^ preprocessor (Ansys, Canonsburg, Pennsylvania, U.S.A.). Fluent^®^ software is used to simulate the fluid flow around the human body, allowing the analysis of values of pressure and speed around (i.e. the human body of a swimmer). With these values we can calculate force components through integration of pressures on the body surface, using a realistic model of a human body, thus decreasing the gap between the experimental and computational data.

The swimmer was modeled as if he were gliding underwater in a streamlined prone position, with hands overlapping, head between the extended arms, feet together and plantar flexed. This is the shape usually adopted after the start and while pushing off from the wall after a turn (Marinho et al., 2009b). The swimmer’s model used for the analysis was 1.90 m tall with head, chest, waist and hip circumferences of 0.58 m, 1.02 m, 0.87 m and 0.93 m, respectively. In the streamlined position, the model had a finger to toe length of 2.40 m.

### Computational Fluid Dynamics model

**Figure 1:**
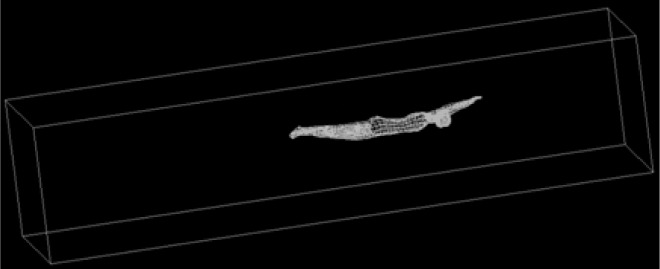
CFD model of the swimmer. The water depth of the model is 2.00 m, with 2.50 m width, and 8.0 m length.

The boundary conditions of the computational fluid dynamics model were designed to represent the geometry and flow conditions of a part of a lane in a swimming pool. The water depth of the model was 2.00 m with 2.50 m width. The length was 8.0 m. The distance between the swimmer and the front surface was 2.0 m and to the back surface was 3.60 m. The swimmer’s model middle line was placed at different water depths between 0 and 1.0m underwater, in 0.25m increments (Figure 1).

The model’s body surface had roughness parameters of zero. The whole domain was meshed with 900 million cells. The grid was a hybrid mesh composed of prisms and pyramids. Significant efforts were conducted to ensure that the model would provide accurate results, namely by decreasing the grid node separation in areas of high velocity and pressure gradients.

Steady-state computational fluid dynamics analyses were performed using the Fluent^®^ code and the drag coefficient and the drag force was calculated for velocities ranging from 1.5 to 2.5 m/s, in increments of 0.5 m/s, which represents the velocity range used by club to elite level swimmers during the push-off and glide following a turn (Blanksby et al., 1996; Lyttle et al., 1999).

The Fluent^®^ code solves flow problems by replacing the Navier-Stokes equations with discretized algebraic expressions that can be solved by iterative computerized calculations. Fluent^®^ code uses the finite volume approach, where the equations are integrated over each control volume. The solutions of the governing system equations are given in each square element of the discretized whole domain. In order to solve the linear system, Fluent^®^ code adopts an Algebraic Multi-Grid (AMG) solver. We used the segregated solver with the standard k-epsilon turbulence model because this turbulence model was shown to be accurate with measured values in previous research (Moreira et al., 2006; Marinho et al., 2011).

We used a turbulence intensity of 1.0% and a turbulence scale of 0.10 m. Water temperature was maintained at 28º C with a density of 998.2 kg/m and a viscosity of 0.001 kg/m/s, to prevent variations in the coefficient of drag associated with different water temperatures (Lyttle et al., 1998). Incompressible flow was assumed.

## Results

**Figure 2:**
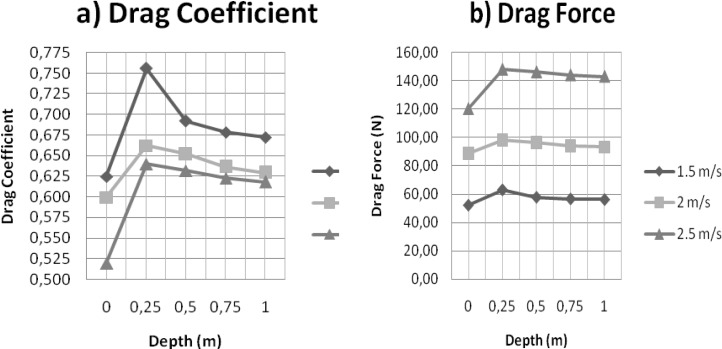
Drag coefficient (a) and drag force (b) as function of depth and velocity.

The drag coefficient and drag forces for the total drag produced by the model, at each of the depths and velocities are listed in Table 1 and presented graphically in Figure 2.

For all the velocities studied (1.5, 2.0 and 2.5m/s), the F_D_ and C_D_ were higher when the glide depth reached 0.25m. From this depth on and as it increases, both F_D_ and C_D_ decreased, remaining almost unchangeable after 0.75 m till 1.0m. The lowest C_D_ and F_D_ values were registered when the swimmer model was gliding at the surface.

For any depth, as the glide velocity of the swimmer model increased, the C_D_ decreased, contrary to what was registered with F_D_, which increased with gliding velocity.

## Discussion

The main purpose of this study was to analyze the effect of depth of glide in the C_D_ and F_D_, using the CFD methodology. The results seem to determine a decrease of drag as the depth of glide increases, although after 0.75 m values remain almost constant.

To accomplish this study a range of depth between 0 and 1.0 m underwater was chosen, since the results obtained by Lyttle et al. (1998) indicate that swimmers should perform their glides at approximately 0.6 m underwater to gain maximum drag reduction benefits. These results (Lyttle et al., 1998) showed a 10–20% decrease in the drag force when travelling at 0.4 and 0.6 m deep relative to gliding at the surface and a 7–14% reduction when gliding at 0.2 m deep.

For all the velocities studied (1.5, 2.0 and 2.5 m/s), the lowest hydrodynamic drag value was registered when the swimmer model was gliding at the surface and the highest occurred when the depth of glide reached 0.25 m. Above this value and as the depth increased, drag values decreased, keeping almost unchangeable after 0.75 m until 1.0 m. This sudden increase of drag, which was registered in the transition of surface glide (C_D_ = 0.625, 0.600, 0.519 to 1.5, 2.0 and 2.5 m/s, respectively) to a 0.25 m underwater glide (C_D_ = 0.756, 0.662, 0.640 to 1.5, 2.0 and 2.5 m/s, respectively) can be due to the fact that, at the surface, part of the swimmer’s body is above the water, showing a smaller frontal surface area, which contributes to the reduction of the pressure drag and, thus, to the reduction of the total drag. Moreover, as the body surface in contact with water is smaller, the friction drag is also reduced (Bixler et al., 2007). This fact is also sustained by Jiskoot and Clarys (1975) who suggested that the combined friction drag and body resistance when immersing the body in the water was greater than the extra wave making resistance resulting from a partially submerged body. However, gliding with half the body emerged is not feasible either after starts or turns, reinforcing the importance of analyzing the underwater glide.

The higher value of hydrodynamic drag at a depth of 0.25 m was the result of a glide made close to the surface, which contributed to the formation of waves at the surface, causing wave drag. Wave drag, together with pressure drag and friction drag have contributed to the increase of total drag (Bixler et al., 2007). Lyttle et al. (1999) reported that there is no significant wave drag when an adult swimmer is gliding at least 0.6 m underwater. A study carried out by Vennell et al. (2006) showed that total drag quickly increases when the body is towed at more shallow depths than above 0.7 m underwater, reaching a 2.4 higher drag than when the body is totally immersed. Wave drag contributes around 50 to 60% to total drag force, in elite swimmers, when swimming at the surface. Moreover, swimmers must be at a depth higher than 1.8 times the diameter of the chest when gliding at a 0.9 m/s velocity, and higher than 2.8 times the diameter of the chest a ta 2.0 m/s gliding velocity, after start and turns so that, a significant wave drag can be avoided. Such conclusions emphasize the importance of reducing wave drag when gliding after starts and turns. In the current study, using a three-dimensional CFD simulation, similar results were obtained, reinforcing the importance of depth position during gliding. A decrease in hydrodynamic drag values as depth increase was verified, although after 0.75 m values remained almost constant, suggesting there is a critical point, beyond which wave drag is almost null (Lyttle et al., 1999; Vennel et al., 2006). This drag reduction due to underwater gliding can lead to improve swimming performance, through an increase in gliding velocity during this phase.

The same tendency regarding the effects of depth on drag was obtained for the three velocities analyzed. However, C_D_ decreased as gliding velocity of the model increased. According to Vogel (1994), a body that moves through a fluid must overcome drag force, which is proportional to C_D_, to the front surface area and to the square of swimming velocity in relation to fluid velocity. When swimmers increase their swimming velocity, they generate higher turbulence and friction, which consequently results in a F_D_ rise, as occurred in the current study (Figure 2). The effects of velocity are so “powerful” that, if doubled, F_D_ is quadrupled. However, as stated by Lavoie and Montpetit (1986) and Vorontsov and Rumyantsev (2000), the body tends to reach a more hydrodynamic position in water with the velocity increase due to the hydrostatical impulse. This impulse reduces the front surface area opposed to displacement and, thus, the C_D_, by the reduction of the relative influence of shape drag.

Some limitation of the current study can be addressed. This analysis was carried out in one swimmer only, thus one should be careful when transferring these data to other swimmers. Although CFD seems to be an interesting tool to examine the water flow around the swimmer’s body and to compute hydrodynamic drag (Bixler and Schloder, 1996), these procedures, especially when three-dimensional models are used, required a lot of time and equipment. Hence, until this moment CFD studies in sports only applied one single digital model during simulations (Marinho et al., 2009c). However, it raises the question if different swimmers would present the same tendency as the one studied in this paper. Moreover, this study only analyzed a passive drag situation, when the swimmer is passively gliding after starts and turns. In the future, the development of this methodology must consider the body movements in the CFD domain, analysing, for instance, the second part of the gliding when the swimmer is kicking, allowing to study the total underwater phase.

As a conclusion, one can state that the water depth seems to have a positive effect on reducing hydrodynamic drag during the gliding. Although increasing depth position could contribute to a decrease in hydrodynamic drag, this reduction seems to be lower with depth, especially after 0.75 m depth, thus suggesting that performing the underwater gliding (and the underwater dolphin kicking) more than 0.75 m depth will not be to the benefit of the swimmer. Nevertheless, a commitment between decreasing drag (by increasing water depth) and gliding distance should be the main concern of swimmers and an important goal to be addressed in future investigations.

## Figures and Tables

**Table 1:** Drag coefficient and drag force values for different velocities and depth during gliding.

	Drag coefficient	Drag Force (N)
Depth = 0 m		
v = 1.5 m/s	0.625	52.04
v = 2.0 m/s	0.600	88.78
v = 2.5 m/s	0.519	120.18

Depth = 0.25 m		
v = 1.5 m/s	0.756	62.94
v = 2.0 m/s	0.662	98.04
v = 2.5 m/s	0.640	148.04

Depth = 0.50 m		
v = 1.5 m/s	0.692	57.64
v = 2.0 m/s	0.652	96.52
v = 2.5 m/s	0.632	146.16

Depth = 0.75 m		
v = 1.5 m/s	0.678	56.51
v = 2.0 m/s	0.636	94.21
v = 2.5 m/s	0.623	144.06

Depth = 1.0 m		
v = 1.5 m/s	0.672	56.01
v = 2.0 m/s	0.629	93.14
v = 2.5 m/s	0.618	142.95
